# Depression, Disability, and Cognitive Impairment Among Elders With Medical Illnesses Attending Follow-Up Clinics at a Tertiary Care Hospital in Northern Sri Lanka

**DOI:** 10.7759/cureus.32379

**Published:** 2022-12-10

**Authors:** Bhavana Sivayokan, Nipuna C Somasiri, Thayananthi Maheswaran, Nihanatha Mahendrarajah, Achala I Gunarathna, Pethirupillai A Coonghe, Navaneethakrishnan Suganthan, Sambasivamoorthy Sivayokan

**Affiliations:** 1 Community and Family Medicine, University of Jaffna, Jaffna, LKA; 2 Medicine, University of Jaffna, Jaffna, LKA; 3 University Medical Unit, Teaching Hospital, Jaffna, LKA; 4 Psychiatry, University of Jaffna, Jaffna, LKA; 5 Mental Health Unit, Teaching Hospital, Jaffna, LKA

**Keywords:** geropsychiatry, gerontology, health disparities, functional decline, depression in elderly, dementia, aging

## Abstract

Introduction: The rising proportion of the elderly is increasingly affected by non-communicable diseases. Despite an abundance of literature suggesting that elders with medical conditions are more vulnerable to depression, disability, and cognitive impairment, these tend to go unnoticed and unaddressed. This study describes the prevalence and correlates of depression, disability, and cognitive impairment among elders with medical illnesses attending follow-up clinics in a tertiary care hospital in northern Sri Lanka.

Methods: This descriptive cross-sectional study was carried out among 122 elders (≥60 years) attending medical clinics at Teaching Hospital Jaffna. Depression, disability, and cognitive impairment were assessed by the 15-item Geriatric Depression Scale, 12-item World Health Organization Disability Assessment Schedule 2.0, and Montreal Cognitive Assessment, respectively. Student’s T-Test, ANOVA, and correlation coefficient were used in analyzing data using Statistical Package for Social Sciences 25 (SPSS-v25) (IBM, New York, United States).

Results: The mean age of the participants was 68.3 years (SD=5.70); 58 (47.5%) were males and 64 (52.5%) were females. Prevalence of depression was 44.3% (95% CI=35.5-53.1), while disability was 95.9% (95% CI=92.4-99.4) and cognitive impairment was 80.3% (95% CI=73.2-87.4). Depression was significantly associated with gender (p=0.013), marital status (p=0.019), and living arrangement (p<0.001). Cognitive impairment was significantly associated with education level (p=0.045), and disability was associated with education level (p=0.008) and marital status (p=0.027). Among the study participants, only 12 (9.8%) had previously sought professional help for depression, disability, or cognitive impairment.

Conclusion: Depression, disability, and cognitive impairment are common among the elderly attending medical clinics in Teaching Hospital Jaffna, and are, in most cases, unaddressed.

## Introduction

The proportion of elderly in the population is rising globally with 22% expected to be in the ≥60 years age group by 2050 [1]. Although conventionally defined as people aged 65 years and above [2], in many low- and middle-income countries (LMICs), including Sri Lanka, people aged 60 years and over are considered to belong to the elderly age group [3] due to differences in pre-defined retirement age, life expectancy, lifestyle, and overall health status. In Sri Lanka, between 2001 and 2019, the proportion of citizens 60 years of age and above rose from 9.9% to 12.3% [4]. By the year 2041, it is estimated that over a quarter (28%) of the country’s population will be above 60 years [5].

With aging comes a multitude of physical and physiological changes, which not only bring new ailments but also aggravate existing medical and psychological problems [6]. A review of the literature revealed that depression and cognitive impairment are among the commonest psychological problems faced by the elderly [1] and that functional decline and disability increased not only with advancing age but also with the presence of co-morbid medical illnesses [7]. In addition, various social factors shape the unique challenges the elderly encounter, including living conditions, loneliness, financial difficulties, their perceived status in the community, lack of psychosocial support, and limited availability of specialized services. Together, these challenges can be detrimental to elders’ health and quality of life [8], the effects of which could be augmented in the presence of a multiplicity of health problems [9].

Little is known about the mental well-being of the elderly in northern Sri Lanka. A multi-center study conducted in the Northern Province [10] found the prevalence of depression among elders attending primary healthcare centers to be 11.6%. A more recent study in the north [11] among users of primary healthcare centers (including the elderly) yielded a much higher prevalence of depression of 41.6% and found old age to be a significant contributing factor. We identified only one published study [12] on cognitive impairment, in a clinical setting, among inward patients in the geriatric age group at a tertiary care center, where the prevalence was found to be 67.4%. Despite a community-based study conducted in southern Sri Lanka [13] over two decades ago yielding a prevalence of disability of 20% among the elderly, and wide recognition that medical illness brings about a decline in functional ability [14], recent studies on disability among the elderly with medical illness are lacking.

Elders in northern Sri Lanka have been through three decades of armed conflict. Home to a predominantly Tamil-speaking population, the region has undergone a noticeable demographic and social transformation during the post-war years due to internal and external displacement, migration, disappearances, and death [15]. The civil war has left many socially and economically unstable due to the loss of property and livelihoods, changed family and community dynamics [15], and eroded community support systems [16]. Migration and death of younger cohorts have resulted in a scarcity of formal and informal caregivers [17]. This protracted conflict has left a great deal of trauma as its legacy, with multiple studies throwing light on its adverse mental health implications [18,19].

In Jaffna, the most populated district in northern Sri Lanka, elders (≥60 years) comprise about 15% of the local population [20]. A recent survey found that 55% of the elderly in Jaffna have a monthly income below the national poverty line [17]. At present, a substantial proportion of elders live alone, with or without their elderly spouses, in a delicate equilibrium. This equilibrium is constantly under threat with various factors including financial constraints, lack of mutual care giving, and loss of loved ones, converging on their physical, mental, and social well-being. In addition, the social isolation and sedentary lifestyle of the elderly in this region may also contribute to a decline in cognitive status [21].

Amid an epidemiological transition, the elderly population in Jaffna is increasingly affected by non-communicable diseases (NCDs). Despite an abundance of literature suggesting that elders with medical conditions are more vulnerable to depression, disability, and cognitive impairment [9,14], potential impacts of medical illnesses on the elderly in the north and elsewhere tend to go unnoticed, even as physical aspects of NCDs are identified and treated. Studies among elders with multiple morbidities in community and clinical settings in other countries reveal that depressive symptoms are frequently missed and untreated [22].

Without a well-established system of family practice [23], elders in Sri Lanka mostly seek medical care in government hospitals, where free health services are available, with a smaller fraction approaching the private sector for healthcare. Given the high levels of poverty in the north, most elders have access to care for medical illnesses only at government hospitals. With bed strength of 1,310, Teaching Hospital Jaffna (THJ) is the largest and only tertiary health care facility with multiple specialties in the Northern Province, catering to many elders with medical problems in Jaffna and other northern districts. The prevalence of depression, disability, and cognitive impairment among elders with medical illnesses in Jaffna is not known. Addressing this gap, this study aims to describe the prevalence and correlates of depression, disability, and cognitive impairment among elderly patients attending the medical clinics at THJ.

## Materials and methods

This institution-based descriptive cross-sectional study was conducted in the medical follow-up clinics at THJ, Sri Lanka, among patients of all genders, aged 60 and above, attending clinics either for the first time or for a follow-up visit. The exclusion criteria included severe communication problems as a result of conditions such as hearing or speech disability, acute or degenerative neurological diseases, active hallucinations, and formal thought disorders.

Data were collected from all patients who met the study criteria during a four-month period (November 2020 to March 2021) in the midst of the COVID-19 pandemic. A total of 166 patients were approached, of whom 122 responded. An interviewer-administered questionnaire designed by the research team was used to collect basic details regarding the participants, including age, gender, religion, ethnicity, educational level, marital status, living arrangement, distance traveled to come to the clinic, illnesses for which they were under follow-up, and whether they had sought professional help for depression, cognitive impairment, or any functional disabilities. The questionnaire also contained the 15-item Geriatric Depression Scale (GDS) [24], 12-item World Health Organization Disability Assessment Schedule 2.0 (WHO DAS 2.0) [25], and the Montreal Cognitive Assessment (MoCA) [26].

The 15-item GDS is a tool that has been tested and extensively used among the elderly and is easy to administer to those with impaired cognition. The 12-item WHO DAS 2.0 [25] is internationally validated and has been used widely. The Montreal Cognitive Assessment (MoCA) possesses high sensitivity and specificity for detecting mild cognitive impairment and has been translated into Tamil, the local language, and validated. The GDS and WHO DAS 2.0 were translated from English to Tamil, and judgmental validation was done by an experienced psychiatrist.

Cut-offs to categorize depression, disability, and cognitive impairment were drawn from the literature. The scores of the Geriatric Depression Scale were categorized as normal (0-4), mild depression (5-8), moderate depression (9-11), and severe depression (12-15) [24]. In the WHO DAS 2.0 scale, each of the 12 items was scored from 0 to 4, where 0, 1, 2, 3, and 4 represented no, mild, moderate, severe, and extreme/complete difficulty, respectively, in the relevant activity; overall scores were categorized as no disability (0), mild disability (1-4), moderate disability (5-9), and severe disability (10-48) [25]. Scores of the MoCA scale were categorized as normal (score ≥ 24) and abnormal (score ≤ 23) [27].

Data were analyzed using Statistical Package for Social Sciences, version 25 (IBM, New York, United States). Prevalence and levels of depression, disability, and cognitive impairment were determined by the scores obtained from the scales, based on the cut-offs. Mean differences in depression, disability, and cognitive impairment scores by sociodemographic factors were measured using independent t-test and ANOVA, as appropriate. Correlations between depression, disability, and cognitive impairment were measured using Pearson’s correlation. Ethics approval was obtained from the Ethics Review Committee of the Faculty of Medicine, University of Jaffna (approval number: ERC/116/6/11).

## Results

Among the 166 elders approached, 122 responded, making the response rate 73.5%. The sociodemographic characteristics of the participants are tabulated in Table 1. Most of them were from Jaffna. The mean age of the participants was 68.3 years (SD=5.70), with a median of 68 years (interquartile range (IQR)=63-72). Among the participants, 58 (47.5%) were males and 64 (52.5%) were females.

**Table 1:** Sociodemographic details of the study participants (N=122) GCE A/L, General Certificate of Education Advanced Level.

Sociodemographic factors	Characteristics	N (122)	%
Age (years)	60-64	38	31.2
	65-69	36	29.5
	70-74	29	23.8
	75-79	17	13.9
	80-84	2	1.6
Gender	Male	58	47.5
	Female	64	52.5
Ethnicity	Sri Lankan Tamil	120	98.4
	Indian Tamil	2	1.6
Religion	Hinduism	96	78.7
	Christianity	25	20.5
	Islam	1	0.8
Educational level	No formal education	4	3.3
	Primary education (grades 1 to 5)	24	19.7
	Secondary education (grade 6 to GCE A/L or equivalent)	71	58.2
	Tertiary education (education beyond school)	23	18.8
Marital status	Unmarried	10	8.2
	In marital life	87	71.3
	Separated	1	0.8
	Divorced	1	0.8
	Widowed	23	18.9
Living arrangement	Living alone	17	13.9
	Living with spouse only	38	31.2
	Living with an unmarried child (with or without spouse)	24	19.7
	Living with extended family	43	35.2
Distance traveled to hospital	5 km	43	35.2
	5-20 km	64	52.5
	>20 km	15	12.3

**Figure 1:**
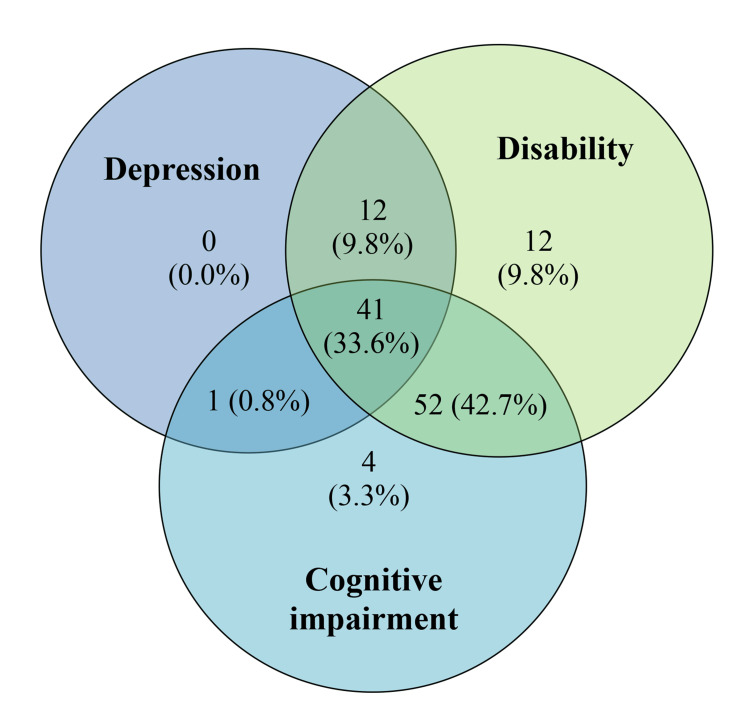
Prevalence of depression, disability, and cognitive impairment among the study participants (N=122)

The prevalence of depression, disability, and cognitive impairment in the sample is given in Table 2. The overall number of participants with depression was 54 (44.3%, 95% CI=35.5-53.1), while those with disability were 117 (95.9%, 95% CI=92.4-99.4) and those with cognitive impairment were 98 (80.3%, 95% CI=73.2-87.4). The coexistence of depression, disability, and cognitive impairment in the sample is shown in Figure 1, indicating that all participants had at least one of the three conditions.

**Table 2:** Prevalence of depression, disability, and cognitive impairment (N=122)

Condition and severity		N	%
Depression			
	Mild depression	32	26.2
	Moderate depression	15	12.3
	Severe depression	07	5.8
	Total	54	44.3
Disability			
	Mild disability	29	23.8
	Moderate disability	21	17.2
	Severe disability	67	54.9
	Total	117	95.9
Cognitive impairment		98	80.3

In the sample, 66 participants (54.1%) were followed up at the medical clinic for one illness, while 40 (32.8%) were followed up for two illnesses and 16 (13.1%) for three or more illnesses. The most common illnesses the participants had were diabetes mellitus (53.3%), followed by hypertension (45.1%), dyslipidemia (21.3%), ischemic heart disease (18.0%), and bronchial asthma (12.3%). Only 12 participants (9.8%) had previously sought professional help for depression, disability, or cognitive impairment.

An analysis of correlations among depression, disability, and cognitive impairment as well as correlation between the number of entities and the number of medical illnesses the participants had elicited interesting results. Depression and cognitive impairment (r=0.232, p=0.010) and depression and disability (r=0.388, p<0.001) were significantly correlated but not cognitive impairment and disability (r=0.166, p=0.068). We also found a significant correlation between the number of medical illnesses for which the participant was followed up at the medical clinic and the number of assessed entities present (r=0.238, p=0.008).

The distribution of the level of depression, disability, and cognitive impairment by sociodemographic factors is available in the Appendix, Tables 6-11. Compared to the proportion of old-elderly (10.5%), the proportion of young-elderly with moderate-severe depression (19.4%) was greater. The prevalence of moderate-severe depression among females (23.4%) was almost double the prevalence among males (12.1%). More than half of those living alone (53.9%) had moderate-severe depression. Over three-fifths (65.8%) of those living with their spouse had no depression. When considering the level of disability, more women (59.4%) than men (50.0%) had severe disability. Cognitive impairment was more prevalent among those with primary education or less (92.9%) compared with elders who had at least secondary education (76.6%).

Inferential analysis showed statistically significant differences in mean GDS scores based on gender, marital status, and living arrangement (Table 3). Mean GDS of males (3.93, SD=3.22) was significantly lower than that of females (5.52, SD=3.70; p=0.013), while the mean GDS score of those in marital life (4.29, SD=3.32) was lower than others (5.94, SD=3.88; p=0.019) and that of those living alone (7.88, SD=3.84) was higher than those living with their spouse (3.63, SD=2.92), unmarried child (5.46, SD=3.43), and extended family (4.14, SD=3.34, p<0.001). Mean WHO DAS 2.0 scores differed statistically by educational level and marital status (Table 4) with mean WHO DAS 2.0 of those having secondary or tertiary education (10.29, SD=8.05) being lower than others (15.00, SD=8.55; p=0.008) and that of those in marital life (10.31, SD=8.06) being lower than others (14.00, SD=8.68; p=0.027). Mean MoCA scores differed significantly by educational level (Table 5), with mean MoCA score of those with secondary or tertiary education (20.33, SD=4.3) higher than others (18.46, SD=3.99).

**Table 3:** GDS scores by sociodemographic factors GDS, Geriatric Depression Scale; SD, standard deviation; MD, mean difference; CI, confidence interval. Higher score indicates a greater level of depression.

Sociodemographic factors	Characteristics	GDS score			T statistic, p value
		Mean	SD	MD and CI	
Age (years)	60-74	4.93	3.63	1.090 (-0.664, 2.844)	t(120)=1.230, p=0.221
	75	3.84	3.06		
Gender	Male	3.93	3.22	-1.585 (-2.834, -0.335)	t(119.8)=−2.528, p=0.013
	Female	5.52	3.70		
Religion	Hinduism	5.05	3.67	1.360 (-0.012, 2.732)	t(48.8)=1.992, p=0.052
	Others	3.69	2.91		
Educational level	No formal education/primary education	5.68	3.96	1.189 (-0.318, 2.696)	t(120)=1.563, p=0.121
	Secondary/tertiary education	4.49	3.40		
Marital status	In marital life	4.29	3.32	-1.656 (-3.039, -0.272)	𝑡(120)=−2.370, p=0.019
	Others	5.94	3.88		
Living arrangement	Alone	7.88	3.84	-	F(3,118)=7.403, p<0.001
	Spouse	3.63	2.92		
	Unmarried child	5.46	3.43		
	Extended family	4.14	3.34		
Distance traveled	5 km	4.91	3.49	-	F(2,119)=1.839, p=0.163
	5-20 km	5.05	3.74		
	>20 km	3.13	2.53		

**Table 4:** WHO DAS 2.0 scores by sociodemographic factors WHO DAS 2.0, World Health Organization Disability Assessment Schedule 2.0; SD, standard deviation; MD, mean difference; CI, confidence interval. Higher score indicates a greater level of disability.

Sociodemographic factors	Characteristics	WHO DAS 2.0 score			Association
		Mean	SD	MD and CI	
Age (years)	60-74	11.31	8.03	−0.374 (−4.530, 3.783)	t(120)=−0.178, p=0.859
	75	11.68	10.32		
Gender	Male	10.59	8.90	−1.492 (−4.498, 1.515)	t(120)=−0.983, p=0.328
	Female	12.08	7.87		
Religion	Hinduism	11.35	8.54	−0.069 (−3.750, 3.612)	t(120)=−0.037, p=0.970
	Others	11.42	7.91		
Educational level	No formal education/primary education	15.00	8.55	4.713 (1.231, 8.195)	t(120)=2.680, p=0.008
	Secondary/tertiary education	10.29	8.05		
Marital status	In marital life	10.31	8.06	−3.690 (−6.955, −0.424)	t(120)=−2.237, p=0.027
	Others	14.00	8.68		
Living arrangement	Alone	11.47	6.91	-	F(3,118)=0.925, p=0.431
	Spouse	10.26	7.81		
	Unmarried child	13.79	10.30		
	Extended family	10.95	8.21		
Distance traveled	5 km	12.07	9.08	-	F(2,119)=1.166, p=0.315
	5-20 km	11.61	8.23		
	>20 km	8.33	6.54		

**Table 5:** MoCA scores by sociodemographic factors MoCA, Montreal Cognitive Assessment; SD, standard deviation; MD, mean difference; CI, confidence interval. Lower score indicates a greater level of cognitive impairment.

Sociodemographic factors	Characteristics	MoCA score			Association
		Mean	SD	MD and CI	
Age (years)	60-74	20.10	4.42	1.255 (–0.886, 3.396)	t(120)=1.160, p=0.248
	75	18.84	3.80		
Gender	Male	20.43	4.19	1.009 (–0.544, 2.562)	t(120)=1.287, p=0.201
	Female	19.42	4.45		
Religion	Hinduism	19.96	4.27	0.266 (–1.640, 2.172)	t(120)=0.276, p=0.783
	Others	19.69	4.66		
Educational level	No formal education/primary education	18.46	3.99	–1.866 (–3.691, –0.040)	t(120)=−2.023, p=0.045
	Secondary/tertiary education	20.33	4.36		
Marital status	In marital life	20.10	4.04	0.703 (–1.018, 2.425)	t(120)=0.809, p=0.420
	Others	19.40	5.04		
Living arrangement	Alone	19.41	5.43	-	F(3,118)=0.151, p=0.929
	Spouse	20.18	3.76		
	Unmarried child	19.67	4.33		
	Extended family	19.98	4.48		
Distance traveled	5 km	19.58	4.34	-	F2,119)=0.538, p=0.585
	5-20 km	19.88	4.31		
	>20 km	20.93	4.59		

Responses to individual questions in the GDS and WHO DAS 2.0, as well as the domain-wise MoCA score, are given in the Appendix, Tables 6-11. When responses to individual GDS questions were analyzed, we observed that most participants (82%) reported being satisfied with life, in good spirits (84%) and happy (84.4%), most of the time during the past week. However, almost two-thirds (65.6%) responded that they prefer to stay at home, and 50.8% felt that they have more problems with memory than most.

With respect to WHO DAS 2.0, while over 90% of participants reported not facing any difficulties with maintaining personal hygiene, a substantial proportion agreed to some level that they had difficulty in standing for long periods (66.4%), walking 1 km or more (65.6%), learning a new task (59.8%), and taking care of household responsibilities (52.5%). Three-quarters of the participants (74.6%) admitted to have been emotionally affected by their health problems.

On analyzing the MoCA scores, we observed that the least number of participants had problems in the Naming and Orientation domains, with only two (1.6%) scoring less than the full score in Naming and 12 (9.7%) scoring less than the full score in Orientation. The domain affected most was the Delayed recall domain, in which only four participants (3.2%) scored the full score. In fact, one-third of the sample (37.7%) scored zero in this domain. Other domains in which participants did not perform well included, Abstraction and Visuospatial/Executive domains, in which 19.7% and 18.05%, respectively, scored zero.

## Discussion

The study sample reflected the age distribution and ethnic makeup of the Jaffna district [20]. Other sociodemographic characteristics, in particular, the lower proportion of old-elderly (≥75 years) (15.5%) in the sample and over three-quarters having at least secondary education (77%) and not living alone (86.1%), are critical to note, as they may have impacted the study findings.

Prevalence of depression, disability, and cognitive impairment

This study revealed that 44.3% of the study participants had depression, with 26.2% having mild depression, 12.3% having moderate depression, and 5.8% having severe depression. This is much higher than the 11.6% recorded among elders in a community-based study conducted in northern Sri Lanka [10], but on par with the 41.6% reported in a more recent study among adults seeking care at primary healthcare institutions in the Northern Province [11]. Studies from South Asia report similar prevalence rates of old-age depression [28,29], while other international studies report lower rates [9,30,31]. In the present study, the higher prevalence of depression may be explained by the fact that our sample, recruited from medical clinics, had multiple comorbid medical conditions [1], as compared with community-based studies [9,30].

Using WHO DAS 2.0, we found very high rates of disability among elders (95.9%), among whom 57.3% had a severe disability, as compared with 20%, the rate of disability reported in an earlier study in Kandy [13]. WHO DAS 2.0 defines disability in terms of cognition, mobility, self-care, getting along, life activities, and participation [25]. The Kandy study measured disability in relation to impairment of activities of daily living (ADLs) and instrumental activities of daily living (IADLs). Several other community-based studies [7,32] also describe a lower prevalence of disability in the elderly. Many of these studies assessed ADLs and IADLs using different scales. However, a study conducted in India [33], which used the WHO DAS 2.0 36-item version, found the prevalence of disability to be similar to our study, with 92%-100% of women and 82%-99% of men having disability.

When the MoCA scores were analyzed, we found that 80.3% of study participants had cognitive impairment. This is higher than the prevalence rate of 67.4% in a prior study among hospitalized elderly patients in Jaffna [12] that used the Concise Cognitive test. It is also higher than the value (>50%) obtained from a hospital-based study in Colombo [34] that used Mini-Mental State Examination (MMSE). While differing scales may explain the lower prevalence identified in these two hospital-based studies, it is interesting to note that the value obtained in the present study was much higher than the prevalence of mild cognitive impairment (20.9%) in a community-based study in Jaffna [35] using MoCA. This strongly suggests that comorbid medical illnesses may negatively impact cognition in the elderly. Other studies which did not explore comorbidities report much lower rates of cognitive impairment [36-38].

Taken together, the prevalence of depression, disability, and cognitive impairment was all higher than the figures obtained from prior studies in the region and country. Indeed, we found that all participants had at least one of the three conditions assessed, depression, disability, and cognitive impairment, and less than 10% of the sample had sought professional help for them. While the panic created in the wake of the COVID-19 pandemic, along with the travel restrictions and resulting social isolation, could have had an impact on the mental well-being of the elderly [39], the question remains whether the pandemic alone could explain the high prevalence rates gleaned from this study or whether they are linked with specific medical or lifestyle-related factors among elders with chronic medical illnesses, a neglected area of research in Sri Lanka and other LMICs.

Correlation between depression, disability, and cognitive impairment

The results of this study indicate that functional disability and cognitive impairment are significantly correlated with depression. Similar correlations were reported in a 2014 study in the Northern Province [10] that explored various parameters among adults visiting primary care facilities. A nation-wide survey in South Korea [40] on risk factors for late-life depression showed that cognitive impairment, as revealed by low MMSE scores, was associated with a higher risk of depression. The Aging, Demographics, and Memory (ADAM) study [41] found that the prevalence of depression was high in those with cognitive impairment when compared to those with normal cognitive status. Similar associations have been reported in other studies around the world [36,42], suggesting that these conditions are very much interlinked and must be approached in tandem.

Differences in sociodemographic factors

In the present study, depression, disability, and cognitive impairment did not differ by age group. Several studies from around the world [38,43,44] suggest that cognitive impairment worsens with age. Although the mean MoCA score of the old-elderly was lower than that of the young-elderly, signaling a higher level of cognitive impairment in the older age group, this difference was not statistically significant (p=0.093). Further, despite studies showing that depression [30] and disability [7] increase with age, mean GDS and WHO DAS 2.0 scores were not statistically different in the young- and old-elderly (p>0.05). These results may be influenced by the makeup of the sample, which comprised very few old-elderly.

Mean GDS score was higher among females than among males (p=0.013). This preponderance of depression among females has also been noted in studies carried out locally and internationally [30,45]. While female preponderance in the prevalence of cognitive impairment has been noted in local and international studies alike [12,44], the present study elicited a slightly lower mean score of MoCA among females than among males, a difference that is not statistically significant (p=0.20). Similarly, although numerous studies show that females are affected more by disability, it was not reflected in this study (p=0.328). These contradictions need further exploration.

Higher levels of educational attainment and cognitive activity are shown to be protective against Alzheimer’s disease, a form of dementia [46]. As mild cognitive impairment is known to have a risk of progressing to dementia [46], factors that have a protective effect against dementia are also protective against cognitive impairment. This was reflected in our study as those with at least secondary education were found to have a higher MoCA mean score, and thus less cognitive impairment, than those with primary education or less (p=0.045). Similar findings are reported in several other settings locally [35,47] and internationally [36,43]. In addition, this study reveals that those with primary education or less had significantly higher WHO DAS 2.0 scores than those with at least secondary education (p=0.008), a result corroborated by a study in a community setting [32]. These differences may be explained by the fact that education level is also a marker of socioeconomic status (SES); disability is known to be associated with SES in the elderly [48]. Although several studies [40,45] have elicited a significant association between educational level and depression, it was not reflected here.

In this study, a significant difference of mean GDS scores (p=0.019) was found according to the participants’ marital status, with those in marital life having a lower GDS score than others. This has been corroborated by other studies [49,50] which reveal a significant association between widowhood and depression. Interestingly, this study also reveals that those in marital life had a significantly lower WHO DAS 2.0 score than others (p=0.027), a finding that is not corroborated in the literature. This might, in part, be due to the fact that most studies used a tool other than WHO DAS 2.0 to assess disability. However, WHO DAS 2.0 measures disability not only in terms of mobility and activity level but also in terms of cognition and psychological aspects such as getting along and participation. As marriage is known to improve psychological well-being [51], that elders in married life had lower levels of disability based on WHO DAS 2.0 is not surprising. Our study did not reveal a statistically significant relationship between cognitive impairment and marital status.

Apart from one’s spouse, the degree of structural support or integration in a social network is said to have a direct positive effect on well-being [52]. This study reveals that those living alone had a significantly higher level of depression when compared to those living with their spouses and/or children (p<0.001), corroborated by a study conducted in South Korea [40]. Similar differences by living arrangement were not identified in relation to disability or cognitive impairment (p>0.05).

Cultural dimensions

Despite over 80% of the sample claiming to be happy and being satisfied with their life in the GDS, over four in 10 participants were found to have depression and around three-fourths of the sample admitted that they had been emotionally affected by their health in the WHO DAS 2.0. Studies show that alexithymia is highly prevalent in South Asian cultures, in which positive emotions are expressed readily, while negative emotions are suppressed [53], perhaps explaining these conflicting results.

On the other hand, such contradictions may be explained by differing understandings and expectations of aging that may prevail in the community. With over four-fifths of study participants having cognitive impairment, and with almost all the participants having issues with delayed recall in MoCA, the study findings support the claim that South Asians tend to view memory loss as part of normal aging [54], which may lead to delayed help-seeking. This may impede early intervention to halt the progress of dementia, ultimately constituting a burden for the patients, their caregivers, and even the healthcare system [54].

As with any study, this research comes with limitations. Due to the sudden escalation of COVID-19 spread and the associated health guidelines, movement restrictions, and change in healthcare practices, the number of patients attending medical clinics fell during the data collection period, compromising the sample size. This study would have benefited from a community-based design, but this was not possible during the pandemic.

## Conclusions

Depression, disability, and cognitive impairment are common among the elderly attending medical clinics in Jaffna, northern Sri Lanka. While these conditions often coexist, they are mostly untreated. The study highlights the need for guidelines and protocols to actively screen for these conditions at medical and, more importantly, primary care facilities, to initiate early intervention to improve quality of life. While research in this area is much needed, the latter should pave way for a comprehensive policy giving due importance to mental health and disability among the elderly in Sri Lanka.

## Appendices

Appendix

**Table 6:** Responses of the participants to the Geriatric Depression Scale, N=122 GDS, Geriatric Depression Scale.

Question No.	GDS question		Yes	No
1	Are you basically satisfied with your life?	N	100	22
		%	82.0	18.0
2	Have you dropped many of your activities and interests?	N	51	71
		%	41.8	58.2
3	Do you feel that your life is empty? (N=121)	N	41	80
		%	33.9	66.1
4	Do you often get bored?	N	56	66
		%	45.9	54.1
5	Are you in good spirits most of the time?	N	102	20
		%	83.6	16.4
6	Are you afraid that something bad is going to happen to you?	N	31	91
		%	25.4	74.6
7	Do you feel happy most of the time?	N	103	19
		%	84.4	15.6
8	Do you feel helpless?	N	34	88
		%	27.9	72.1
9	Do you prefer to stay at home, rather than going out and doing new things?	N	80	42
		%	65.6	34.4
10	Do you feel you have more problems with memory than most?	N	62	60
		%	50.8	49.2
11	Do you think it is wonderful to be alive now?	N	111	11
		%	91.0	9.0
12	Do you feel pretty worthless the way you are now?	N	28	94
		%	23.0	77.0
13	Do you feel full of energy?	N	82	40
		%	67.2	32.8
14	Do you feel that your situation is hopeless?	N	35	87
		%	28.7	71.3
15	Do you think that most people are better off than you are?	N	51	71
		%	41.8	58.2

**Table 7:** Responses of the participants to the World Health Organization Disability Assessment Schedule, N=122 WHO DAS, World Health Organization Disability Assessment Schedule.

Question No.	WHO DAS 2.0 Questions		None	Mild	Moderate	Severe	Extreme or cannot do
1	Standing for long periods such as 30 minutes?	N	41	12	20	23	26
		%	33.6	9.8	16.4	18.9	21.3
2	Taking care of your house hold responsibilities?	N	58	25	13	20	6
		%	47.5	20.5	10.7	16.4	4.9
3	Leaning a new task, for example, leaning how to get to a new place?	N	49	16	20	25	12
		%	40.2	13.1	16.4	20.5	9.8
4	How much of a problem did you have joining in community activities?	N	55	30	16	15	6
		%	45.1	24.6	13.1	12.3	4.9
5	How much have you been emotionally affected by your health problems?	N	31	33	38	18	2
		%	25.4	27.1	31.1	14.8	1.6
6	Concentrating on doing something for 10 minutes?	N	73	16	10	12	11
		%	59.9	13.1	8.2	9.8	9.0
7	Walking a long distance such as a kilometer (or equivalent)	N	42	15	13	21	31
		%	34.4	12.3	10.7	17.2	25.4
8	Washing your whole body?	N	112	2	1	6	1
		%	91.9	1.6	0.8	4.9	0.8
9	Getting dressed?	N	114	2	0	5	1
		%	93.5	1.6	0	4.1	0.8
10	Dealing with people you do not know?	N	99	9	8	4	2
		%	81.1	7.4	6.6	3.3	1.6
11	Maintaining a friendship?	N	105	9	6	2	0
		%	86.1	7.4	4.9	1.6	0.0
12	Your day-to-day work/school?	N	76	22	14	7	3
		%	62.3	18.0	11.5	5.7	2.5

**Table 8:** Scores of the participants, N=122, for each domain of the Montreal Cognitive Assessment (MoCA) *Maximum score that can be obtained for that domain.

MoCA domain and maximum possible score		0	1	2	3	4	5	6
Visuospatial/executive (5)*	N	22	22	18	25	25	10	-
	%	18.0	18.0	14.8	20.5	20.5	8.2	
Naming (3)*	N	0	1	1	120	-	-	-
	%	0	0.8	0.8	98.4			
Attention (6)*	N	0	3	13	19	30	22	35
	%	0.0	2.4	10.7	15.6	24.6	18.0	28.7
Language (3)*	N	10	30	52	30	-	-	-
	%	8.2	24.6	42.6	24.6			
Abstraction (2)*	N	24	51	47	-	-	-	-
	%	19.7	41.8	38.5				
Delayed recall (5)*	N	46	23	19	20	10	4	-
	%	37.7	18.9	15.6	16.4	8.2	3.2	
Orientation (6)*	N	1	0	1	2	2	6	110
	%	0.8	0.0	0.8	1.6	1.6	4.9	90.3

**Table 9:** Levels of depression by sociodemographic factors

Sociodemographic factors	Characteristics	No depression		Mild depression		Moderate-severe depression	
		N	%	N	%	N	%
Age (years)	60-74 (n=103)	56	54.4	27	26.2	20	19.4
	75 (n=19)	12	63.2	5	26.3	2	10.5
Gender	Male (n=58)	36	62.1	15	25.8	7	12.1
	Female (n=64)	32	50.0	17	26.6	15	23.4
Religion	Hinduism (n=96)	49	51.1	27	28.1	20	20.8
	Others (n=26)	19	73.1	5	19.2	2	7.7
Educational level	No formal education/primary education (n=28)	15	53.6	5	17.8	8	28.6
	Secondary/tertiary education (n=94)	53	56.4	27	28.7	14	14.9
Marital status	In marital life (n=87)	52	59.8	24	27.6	11	12.6
	Others (n=35)	16	45.7	8	22.9	11	31.4
Living arrangement	Alone (n=17)	4	23.6	4	23.5	9	52.9
	Spouse (n=38)	25	65.8	11	28.9	2	5.3
	Unmarried child (n=24)	11	45.9	8	33.3	5	20.8
	Extended family (n=43)	28	65.1	9	20.9	6	14.0
Distance travelled	5 km (n=43)	25	58.1	10	23.3	8	18.6
	5-20 km (n=64)	32	50.0	18	28.1	14	21.9
	>20 km (n=15)	11	73.3	4	26.7	0	0.0

**Table 10:** Levels of disability by sociodemographic factors

Sociodemographic factors	Characteristics	No disability		Mild-moderate disability		Severe disability	
		N	%	N	%	N	%
Age (years)	60-74 (n=103)	3	2.9	43	41.8	57	55.3
	75 (n=19)	2	10.5	7	36.9	10	52.6
Gender	Male (n=58)	4	6.9	25	43.1	29	50.0
	Female (n=64)	1	1.6	25	39.0	38	59.4
Religion	Hinduism (n=96)	5	5.2	38	39.6	53	55.2
	Others (n=26)	0	0.0	12	46.2	14	53.8
Educational level	No formal education/primary education (n=28)	2	7.1	5	17.9	21	75.0
	Secondary/tertiary education (n=94)	3	3.2	45	47.9	46	48.9
Marital status	In marital life (n=87)	4	4.6	40	46.0	43	49.4
	Others (n=35)	1	2.8	10	28.6	24	68.6
Living arrangement	Alone (n=17)	1	5.9	6	35.3	10	58.8
	Spouse (n=38)	3	7.9	16	42.1	19	50.0
	Unmarried child (n=24)	0	0.0	9	37.5	15	62.5
	Extended family (n=43)	1	2.3	19	44.2	23	53.5
Distance travelled	5 km (n=43)	1	2.3	19	44.2	23	53.5
	5-20 km (n=64)	3	4.7	24	37.5	37	57.8
	>20 km (n=15)	1	6.6	7	46.7	7	46.7

**Table 11:** Cognitive status by sociodemographic factors

Sociodemographic factors	Characteristics	Normal cognition		Cognitive impairment	
		N	%	N	%
Age (years)	60-74 (n=103)	23	22.3	80	77.7
	75 (n=19)	1	5.3	18	94.7
Gender	Male (n=58)	11	19.0	47	81.0
	Female (n=64)	13	20.3	51	79.7
Religion	Hinduism (n=96)	19	19.8	77	80.2
	Others (n=26)	5	19.2	21	80.8
Educational level	No formal education/primary education (n=28)	2	7.1	26	92.9
	Secondary/tertiary education (n=94)	22	23.4	72	76.6
Marital status	In marital life (n=87)	17	19.5	70	80.5
	Others (n=35)	7	20.0	28	80.0
Living arrangement	Alone (n=17)	4	23.5	13	76.5
	Spouse (n=38)	6	15.8	32	84.2
	Unmarried child (n=24)	4	16.7	20	83.3
	Extended family (n=43)	10	23.3	33	76.7
Distance travelled	5 km (n=43)	7	16.3	36	83.7
	5-20 km (n=64)	12	18.8	52	81.2
	>20 km (n=15)	5	33.3	10	66.7
